# An Overview of the Epidemiology of Multidrug Resistance and Bacterial Resistance Mechanisms: What Solutions Are Available? A Comprehensive Review

**DOI:** 10.3390/microorganisms13092194

**Published:** 2025-09-19

**Authors:** Victoria Birlutiu, Rares-Mircea Birlutiu

**Affiliations:** 1 Faculty of Medicine, Lucian Blaga University of Sibiu, 550169 Sibiu, Romania; 2 County Clinical Emergency Hospital, 550245 Sibiu, Romania; 3 Department 14-Orthopedics, Anaesthesia Intensive Care Unit, Faculty of Medicine, “Carol Davila” University of Medicine and Pharmacy, 020021 Bucharest, Romania; 4 Foisor Clinical Hospital of Orthopedics, Traumatology, and Osteoarticular TB, 030167 Bucharest, Romania

**Keywords:** antimicrobial resistance, multidrug-resistant bacteria, extensively drug-resistant pathogens, pandrug resistance, bacterial resistance mechanisms, global epidemiology, novel antibiotics

## Abstract

Antimicrobial resistance has emerged as one of the most critical public health challenges of the 21st century, threatening to undermine the foundations of modern medicine. In 2019, bacterial infections accounted for 13.6% of all global deaths, with more than 7.7 million fatalities directly attributable to 33 bacterial pathogens, most prominently *Staphylococcus aureus*, *Streptococcus pneumoniae*, *Escherichia coli*, *Klebsiella pneumoniae*, and *Pseudomonas aeruginosa*. Resistance mechanisms are multifactorial, encompassing enzymatic degradation, target modification, efflux pump overexpression, reduced membrane permeability, and biofilm formation, often in combination, leading to multidrug-resistant, extensively drug-resistant, and pandrug-resistant phenotypes. Alarmingly, projections estimate that by 2050 AMR could result in over 10 million deaths annually. This comprehensive review synthesizes global epidemiological data, insights into bacterial resistance mechanisms, and emerging therapeutic solutions, including novel antibiotics such as lasso peptides and macrocyclic peptides (e.g., zosurabalpin), naturally derived compounds (e.g., corallopyronin, clovibactin, chlorotonil A), and targeted inhibitors (e.g., Debio 1453 for *Neisseria gonorrhoeae*). Addressing the AMR crisis requires coordinated international efforts, accelerated drug discovery, and the integration of innovative non-antibiotic approaches to preserve the efficacy of existing therapies and ensure preparedness against future bacterial threats.

## 1. Introduction

Antimicrobial resistance (AMR) represents a defining global health crisis of our time, severely decreasing the effectiveness of antibiotics that are foundational to modern medicine. Analyses estimate that in 2019, nearly 4.95 million deaths were associated with bacterial AMR, of which approximately 1.27 million were directly attributable to resistant infections [1,2]. These figures underscore that AMR already surpasses the mortality burdens of HIV/AIDS, malaria, and tuberculosis in many regions [3]. Without rapid intervention, annual deaths could climb to 10 million by 2050 [2,3,4,5].

Among bacterial pathogens, the greatest concern is represented by the ESKAPEE group, an acronym encompassing *Enterococcus faecium*, *Staphylococcus aureus*, *Klebsiella pneumoniae*, *Acinetobacter baumannii*, *Pseudomonas aeruginosa*, *Enterobacter* spp., and *Escherichia coli* [6].

The World Health Organization (WHO)’s updated 2024 Bacterial Priority Pathogen List categorizes key resistant pathogens into *critical*, *high*, and *medium* priorities to guide research, resource allocation, and public health strategies [7,8,9]. Pathogens such as carbapenem-resistant *Acinetobacter baumannii*, extended-spectrum β-lactamase-producing *Enterobacterales*, methicillin-resistant *Staphylococcus aureus*, and fluoroquinolone-resistant *Salmonella Typhi* exemplify the organisms that currently defy standard treatments and fuel the AMR crisis [8].

From the mechanism point of view, bacterial resistance is multifactorial. Common pathways include the following: enzymatic degradation of antimicrobials (e.g., β-lactamases), target modification (such as mutations in penicillin-binding proteins or topoisomerases), efflux pump overexpression, porin alterations, and biofilm formation that shelter bacteria from drug exposure [10].

Countering AMR requires not only understanding its epidemiology and molecular underpinnings but also identifying and implementing innovative therapeutic and preventive strategies. Investigational agents offer promise, yet a broader framework incorporating rapid diagnostics, vaccine development, and global surveillance and stewardship policies is essential to regain control over resistant pathogens.

This comprehensive review synthesizes recent data on the global burden of AMR, elaborates on the diverse mechanisms underpinning resistance, and explores the spectrum of emerging therapeutic options. By integrating epidemiological findings with mechanistic insights and forward-looking solutions, we aim to provide a clear and actionable map for clinicians, researchers, and public health professionals committed to addressing this increasing threat.

## 2. Materials and Methods

For this comprehensive review, a literature search was conducted in three major databases, PubMed, Scopus, and Web of Science, to identify studies reporting on the global epidemiology of bacterial infections, mechanisms of antimicrobial resistance, and emerging therapeutic strategies, covering the period from January 1990 to June 2025 using combinations of controlled vocabulary (MeSH terms) and free-text keywords. No restrictions were applied with respect to study design, and both clinical and experimental studies were eligible for inclusion. Only articles published in English and involving human bacterial pathogens were considered. The following search string was used in PubMed and adapted to Scopus and Web of Science: (“antimicrobial resistance”[Mesh] OR “antibiotic resistance”[tiab] OR “multidrug-resistant”[tiab] OR MDR[tiab] OR XDR[tiab] OR PDR[tiab]) AND (“resistance mechanisms”[tiab] OR “enzymatic degradation”[tiab] OR “target modification”[tiab] OR “efflux pump”[tiab] OR “biofilm”[tiab]) AND (“novel antibiotics”[tiab] OR “emerging therapies”[tiab] OR “alternative therapies”[tiab] OR “bacteriophage”[tiab] OR “peptide antibiotic”[tiab]) AND (1990:2025[pdat]) AND english[la]NOT (animals[mh] NOT humans[mh]). Equivalent Boolean queries were constructed for Scopus (TITLE-ABS-KEY) and Web of Science (TS field).

We included studies published in English between January 1990 and June 2025 that addressed the epidemiology of antimicrobial resistance, bacterial resistance mechanisms, or emerging therapeutic strategies. Eligible studies comprised both clinical and experimental research, including in vitro or in vivo investigations, as well as narrative and systematic reviews that provided relevant insights into human bacterial pathogens or clinically significant experimental models. Studies were excluded if they were non-English publications, conference abstracts without full-text availability, reports focused exclusively on fungal, viral, or parasitic resistance, or articles dealing solely with veterinary pathogens without direct human health relevance.

All identified records were imported into a reference management software (Mendeley Reference Manager (Elsevier version v2.138.0) and screened for duplicates. Titles and abstracts were independently reviewed by both authors to assess relevance, and the full texts of potentially eligible studies were retrieved for further evaluation. Data extraction was performed independently by both authors using a structured template, capturing information on epidemiological burden, priority pathogens, resistance determinants, and innovative therapeutic or preventive strategies.

**Figure 1:**
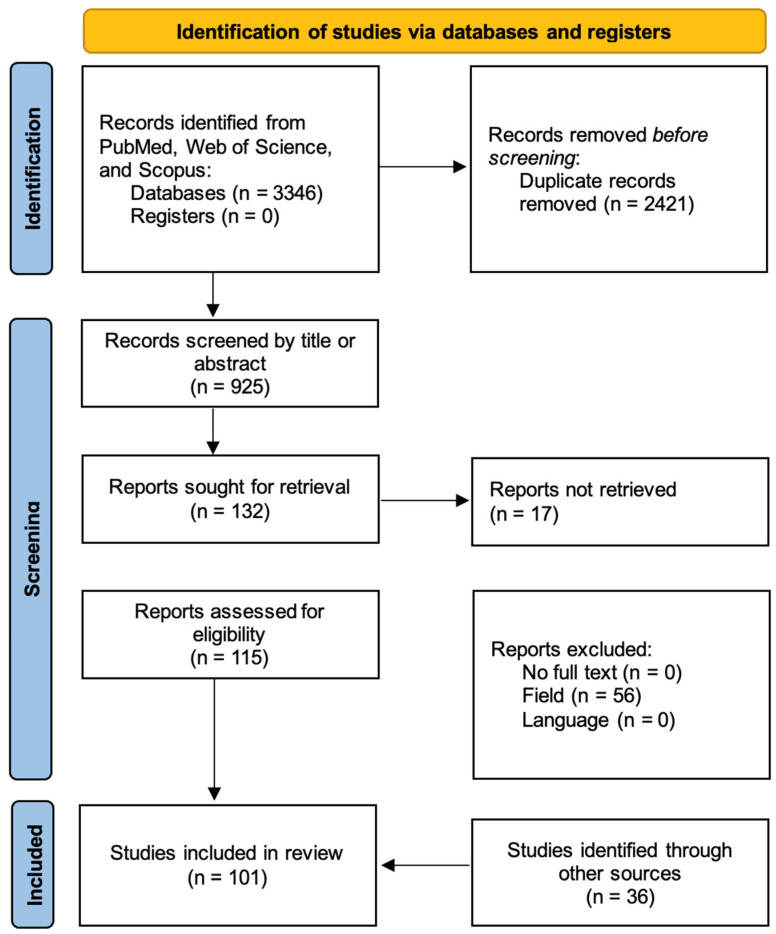
Flow diagram of included studies in the article.

Although no formal risk-of-bias assessment tool was applied due to the expected predominance of narrative and descriptive studies, each article was assessed qualitatively with attention to methodological rigor, transparency of outcome reporting, and potential sources of confounding. The study selection process is summarized in a flow diagram (Figure 1) similar to [11].

## 3. Discussion

### 3.1. Epidemiology and Mechanisms of Resistance

The alarming rise in antibiotic resistance rates, the increasing risk of infections in immunocompromised patients, and the widespread use of invasive diagnostic and therapeutic methods globally are responsible for a staggering number of deaths attributable to bacterial infections each year. In 2019, there were 13.7 million infection-associated deaths, of which 7.7 million were attributable to 33 bacterial pathogens. These accounted for 13.6% of all global deaths and 56.2% of deaths due to sepsis [12].

The most important bacterial pathogens associated with mortality were *Staphylococcus aureus*, a major cause of infections reported across 135 countries and responsible for more than 1 million deaths, particularly among individuals older than 15 years; *Streptococcus pneumoniae*, most frequently linked to deaths in the pediatric population under 5 years; *Salmonella enterica* serovar Typhi, associated with deaths in the 5–14 year age group; and *Escherichia coli*, *Klebsiella pneumoniae*, and *Pseudomonas aeruginosa* (each of the three Gram-negative bacilli being responsible for more than 500,000 deaths in 2019) [12].

In the same year, three major infectious syndromes were predominantly associated with mortality: lower respiratory tract infections (primarily caused by *S. pneumoniae*) and sepsis (dominated by *S. aureus*), each responsible for over 2 million deaths, as well as intra-abdominal infections most frequently caused by *E. coli* (resulting in over 1 million deaths). The highest infection-related mortality rate was recorded in Central Africa, at 394 deaths per 100,000 population, whereas the lowest was documented in Iceland, with 35.7 deaths per 100,000 population [12].

To these figures must be added the growing concern over the escalation of global antimicrobial resistance, which is leaving increasingly limited therapeutic resources for the management of bacterial infections. The concept of multidrug resistance (MDR) refers to resistance against at least three classes of antibiotics, while extensive drug resistance (XDR) is defined as resistance to at least one agent in all but two or fewer antimicrobial classes, and pandrug resistance (PDR) refers to resistance to all agents in all antimicrobial classes [13]. By 2050, it is estimated that antimicrobial resistance will be responsible for more than 10 million deaths annually, prompting the WHO to have released, on 17 May 2024, a list of 15 priority families of pathogens requiring surveillance and urgent therapeutic solutions. These pathogens were stratified into critical, high, and medium priority groups [8]. The critical priority group includes *Acinetobacter baumannii* (carbapenem-resistant) and *Enterobacterales* resistant to third-generation cephalosporins and/or carbapenems. These organisms are characterized by extremely limited or even absent therapeutic options, high morbidity and mortality, rapidly increasing resistance trends, and transmission mechanisms that are difficult to control. Their resistance determinants have been reported globally, and preventive strategies remain limited. The high priority group comprises *Salmonella Typhi* (fluoroquinolone-resistant), *Shigella* spp. (fluoroquinolone-resistant), *Enterococcus faecium* (vancomycin-resistant), *Pseudomonas aeruginosa* (carbapenem-resistant), non-typhoidal *Salmonella* (fluoroquinolone-resistant), *Neisseria gonorrhoeae* (third-generation cephalosporin- and/or fluoroquinolone-resistant), and *Staphylococcus aureus* (methicillin-resistant). These pathogens are also highly challenging to treat, associated with elevated morbidity and mortality, demonstrate alarming upward trends in resistance, spread rapidly, and have only a few available or pipeline therapeutic options. Finally, the medium priority group, which has a significant impact on global health—particularly among vulnerable populations in low-resource settings—includes group A and group B streptococci, *Streptococcus pneumoniae*, and *Haemophilus influenzae*. Against these pathogens, moderate therapeutic resources remain available. They are associated with intermediate levels of morbidity and mortality, exhibit moderate increases in resistance, and can be more effectively controlled through preventive and transmission-reduction strategies. Moreover, both current and near-future treatment options are available for these organisms [8].

In Europe, data provided by the European Centre for Disease Prevention and Control (ECDC) indicate an increasing trend in resistance to third-generation cephalosporins and carbapenems among *Klebsiella pneumoniae* (with carbapenem resistance exceeding 25% in 33% of reporting countries) and *Escherichia coli*. Carbapenem resistance was also common among *Acinetobacter baumannii* and *Pseudomonas aeruginosa*, particularly in countries from Southern and Eastern Europe [14]. For *E. coli* isolates obtained in 2021 from blood and urine cultures in Northern European countries such as Finland and Norway, resistance to fluoroquinolones and third-generation cephalosporins remained below 10%. By contrast, resistance rates in Russia, Turkey, North Macedonia, and Cyprus exceeded 50% [14]. In the same period, carbapenem resistance among *E. coli* isolates was reported at below 1% in eight European countries. For *K. pneumoniae* isolated from blood, urinary, and respiratory infections, resistance rates to third-generation cephalosporins remained below 10% in Northern European countries (Iceland, Norway, Sweden, Switzerland, Denmark, Finland, and Austria) but exceeded 50% in Eastern and Southern Europe. Carbapenem resistance was below 1% in 14 countries, whereas 15 countries reported resistance rates of ≥25%, and eight countries—Romania, Moldova, Serbia, Russia, Ukraine, Georgia, Belarus, and Greece—documented resistance exceeding 50%. For *P. aeruginosa*, most frequently isolated from healthcare-associated infections in immunocompromised patients, carbapenem resistance was below 5% in Denmark and Finland but exceeded 50% in Moldova, Serbia, Ukraine, Russia, Georgia, and Belarus. The highest resistance levels were reported for *A. baumannii*, isolated from blood cultures, ventilator-associated respiratory infections, and wound infections, with resistance reaching at least 50% in 25 European countries. Among *S. aureus* isolates from Europe in 2021, there was a marked increase in community-acquired MRSA, representing ≥25% of isolates in 13 countries, including Romania, Italy, Turkey, and Ukraine. An increasing trend in penicillin resistance among *Streptococcus pneumoniae* strains was observed in several countries, including Romania, France, Serbia, Turkey, and Belarus, where resistance rates were ≥25%. For *Enterococcus faecium*, vancomycin resistance was <1% in France, the Netherlands, Norway, Luxembourg, Sweden, and Finland in 2021, but exceeded 50% in Malta, Cyprus, North Macedonia, Serbia, and Lithuania; in addition, 17 other European countries reported resistance rates above 25% [14].

Most bacterial pathogens exhibit multiple acquired resistance mechanisms against the same antimicrobial agent. The most relevant mechanisms include enzymatic or chemical inactivation, target modification (such as alterations in penicillin-binding proteins, PBPs), reduced permeability through porin loss, overexpression of efflux pumps, and the continuous identification of new resistance gene families [15,16]. These mechanisms add to the background of intrinsic resistance already present in many organisms. Gram-positive bacteria, lacking an outer membrane, generally display higher permeability, which allows the entry of certain antibiotics that are unable to cross the double membrane of Gram-negative bacteria. In Gram-negatives, resistance is further enhanced by porin loss and by modifications in fatty acid and phospholipid content, both of which reduce antibiotic penetration. At the level of porins, antibiotic entry occurs by passive rather than active diffusion [17], and is influenced by pore size and the molecular weight of the antibiotic. Non-specific porins such as OmpF and OmpC facilitate easier passage of antibiotics compared to substrate-specific porins such as PhoE and LamB, which allow permeation primarily for molecules with molecular weights below 600 Da. Structural modifications of OmpK35 and OmpK36 have been shown to underlie carbapenem resistance in *Klebsiella pneumoniae* [18].

Structural modifications of OmpC have also been identified in *Escherichia coli* strains, conferring resistance to cephalosporins, carbapenems, and aminoglycosides [19]. Porins likewise play a critical role in resistance among *Pseudomonas aeruginosa* and *Acinetobacter baumannii*, permitting entry of molecules smaller than 200 Da, while rendering the outer membrane impermeable to larger, hydrophilic antibiotics [20,21,22]. Moreover, synergy between multiple resistance mechanisms and complete porin loss in *P. aeruginosa* effectively prevents the intracellular penetration of antibiotics [23].

Efflux pumps represent another major resistance determinant. These transmembrane proteins extrude diverse substances, including antibiotics, from the periplasmic space. Of the six efflux pump families, the resistance–nodulation–division (RND) family is the most significant, being a key driver of multidrug resistance in Gram-negative bacteria [24,25].

The emergence of MDR frequently involves the interplay of several resistance mechanisms. For instance, increased antibiotic efflux in *Enterobacterales* may be associated with deletion or inhibition of the *acrAB* gene, leading to reduced expression of specific porins such as OmpF and consequently diminished intracellular antibiotic penetration [26]. In *K. pneumoniae*, plasmid-mediated resistance has been shown to enhance the transcription of efflux-associated genes, thereby contributing further to drug resistance [27].

Modification of the cellular target reduces antibiotic efficacy. For fluoroquinolones, for example, structural changes in topoisomerase caused by amino acid substitutions impair drug binding [28]. Genetic alterations leading to structural changes in penicillin-binding proteins (PBPs) are similarly associated with reduced β-lactam activity. A recent example is the modification of PBP3 in *Escherichia coli*, which confers resistance to aztreonam/avibactam [29]. Ribosomal RNA methylation is another mechanism, conferring resistance to macrolides, streptogramins, and lincosamides [30], as well as to aminoglycosides through the action of 16S rRNA methyltransferases [31].

Structural modifications of lipopolysaccharide (LPS) represent the major mechanism of resistance to colistin in Gram-negative bacteria [32,33]. This occurs primarily through the activity of phosphoethanolamine (pEtN) transferases or *mcr* (mobile colistin resistance) genes, explaining the rapid emergence of colistin resistance [34].

For quinolones, high-level resistance often arises from the combination of multiple mechanisms. For instance, acquisition of a *qnr* gene, which encodes Qnr proteins that protect topoisomerases from quinolone action, coupled with chromosomal mutations in *gyrA*, results in significant resistance [35].

Recently, novel resistance mechanisms have been identified in *Staphylococcus aureus* affecting lincosamides, streptogramins, and pleuromutilins (e.g., lefamulin, a representative of the class). These involve Sal-type ABC-F proteins, which bind to 23S rRNA and displace pleuromutilins previously bound to their target [36].

Another key mechanism of resistance is antibiotic inactivation, either by degradation or structural modification via the introduction of new chemical groups. The most prominent example is the hydrolysis of the amide bond in the β-lactam ring by β-lactamases [37]. To date, more than 7000 distinct β-lactamase variants have been identified [38]. According to the Ambler classification, they are grouped into four classes: A, C, and D (serine β-lactamases) and B (zinc-dependent metallo-β-lactamases) [37,39]. Extended-spectrum β-lactamases (ESBLs) are responsible for resistance to broad-spectrum cephalosporins and monobactams [40].

In recent years, carbapenemases have emerged as a particularly concerning problem. These enzymes are classified as KPC (class A), NDM (class B), and OXA (class D), and their rapid global dissemination has drastically reduced therapeutic options, especially in critically ill patients [41,42]. Another mechanism contributing to carbapenem resistance is the combination of porin loss with the presence of extended-spectrum β-lactamases (ESBLs). Notably, NDM carbapenemases confer resistance to nearly all β-lactams, apart from aztreonam [41,42].

Enzymatic inactivation also plays a role in tetracycline resistance, most frequently through the action of Tet(X), which is transmissible both horizontally and via transposable elements. The presence of Tet(X3), Tet(X4), and Tet(X5) has been associated with resistance not only to tetracycline itself but also to newer derivatives such as tigecycline, eravacycline, and omadacycline. Clinical and epidemiological reports have confirmed such cases in *Enterobacterales* and *Acinetobacter* isolates in China [43,44,45].

A major resistance mechanism involves chemical group transfer, whereby antibiotics are inactivated through structural modification. Acetylaminotransferases and phosphotransferases can inactivate aminoglycosides, macrolides, lincosamides, phenicols, and streptogramins A. Recently, ApmA, an acetyltransferase capable of inactivating apramycin, was described, raising concerns regarding potential resistance even to newer aminoglycosides [46].

Rifamycins may be inactivated through enzymatic modifications mediated by ADP-ribosyltransferases, reported in *Mycobacterium smegmatis*, *M. abscessus*, and *M. tuberculosis* [47,48]. Additional inactivation mechanisms include phosphotransferases, glycosyltransferases, and monooxygenases [48,49,50].

Target bypass represents another strategy, whereby bacteria acquire an alternative binding pathway unaffected by the antibiotic. For example, *Staphylococcus aureus* acquires PBP2a encoded by the *mecA* gene [51], while *E. coli* can bypass D,D-transpeptidase PBP5, resulting in β-lactam resistance while maintaining carbapenem susceptibility [52]. Similarly, *Enterococcus* acquires the *vanA* gene, leading to synthesis of modified peptidoglycan precursors that replace the canonical D-alanine–D-alanine terminus with D-alanine–D-serine or D-alanine–D-lactate, thereby conferring vancomycin resistance [53,54]. Acquisition of the Tn1546 transposon from enterococci has enabled the emergence of vancomycin-resistant *S. aureus* (VRSA), first reported in the United States in 2002 [55] and later in Europe [56].

Since 1981, β-lactamase inhibitors have been developed to restore β-lactam activity by forming inhibitor–β-lactamase complexes, in some cases with mild intrinsic antibacterial activity (e.g., sulbactam against *Acinetobacter baumannii*). However, widespread bacterial resistance has also emerged against these combinations, including those paired with newer inhibitors such as vaborbactam, avibactam, relebactam, and durlobactam. For Gram-negative bacilli, polymyxins such as colistin—which increase bacterial membrane permeability—can be combined with other agents to potentiate antibacterial effects, providing a therapeutic option in selected scenarios [57].

The role of biofilm formation on medical devices cannot be overlooked. Biofilms are present on virtually all implanted or indwelling devices—including joint endoprostheses, cochlear implants, ocular implants, breast implants, ureteral and biliary stents, cardiac pacemakers, prosthetic heart valves, in cystic fibrosis airways, ventricular shunts, central venous catheters, tracheal cannulas, and peripheral ulcers—where they profoundly impair antibiotic activity [58,59,60,61]. While planktonic bacteria can be relatively easily eradicated, the penetration of antibiotics into the deeper layers of a biofilm is markedly reduced. This impaired efficacy is further compounded by decreased cellular metabolism, reduced bacterial replication (the stage at which β-lactams are most active), and reduced oxygen availability, which diminishes the activity of oxygen-dependent antibiotics [62].

Additional protective mechanisms within biofilms include the presence of persister cells, especially those with low ATP levels [17], and dormant variants such as cell wall-deficient L-forms, which are structurally deficient and therefore resistant to β-lactam activity [63,64,65]. Biofilm formation is thus considered a distinct pharmacological compartment [66,67].

Furthermore, some β-lactam antibiotics are affected by the inoculum effect (that is defined as the phenomenon of attenuated antibacterial activity at inocula above those utilized for antibiotic susceptibility testing), leading to reduced efficacy. This phenomenon has been described for ampicillin/sulbactam and piperacillin/tazobactam, and to a lesser extent for oxacillin, cephalosporins, and meropenem [68]. Overall, very few antibiotics exhibit clinically relevant activity within biofilms; notable examples with demonstrated penetrative ability include rifampicin, teicoplanin, and fusidic acid.

Currently, the use of β-lactam/β-lactamase inhibitor combinations, such as ceftazidime/avibactam, ceftolozane/tazobactam, meropenem/vaborbactam, imipenem/relebactam, aztreonam/avibactam, or cefiderocol, represents essential therapeutic options for infections caused by carbapenem-resistant Gram-negative bacteria, which have been included by the World Health Organization in the reserve group of antibiotics [8]. Access to these agents, however, varies considerably across European countries, being strongly influenced by economic level and national health policies [69,70].

Phase 3 clinical trials evaluating the comparative efficacy of different therapeutic regimens against carbapenem-resistant Gram-negative bacteria have generally enrolled relatively small numbers of patients, with efficacy conclusions most often expressed in terms of non-inferiority compared to the standard of care. Nevertheless, some trials have reported superiority outcomes, particularly in the treatment of carbapenem-resistant Enterobacteriaceae infections in hospital-acquired pneumonia, ventilator-associated pneumonia, or bloodstream infections, where plazomicin and meropenem or tigecycline demonstrated superior efficacy compared to colistin plus meropenem or tigecycline [71]. Similarly, non-inferiority and, in some cases, superiority, were demonstrated with meropenem–vaborbactam compared to piperacillin–tazobactam in the TANGO I trial of complicated urinary tract infections [72].

Comparable trials have also been conducted for Gram-positive pathogens. For example, multiple phase 3 studies comparing tedizolid with linezolid or standard of care in acute bacterial skin and skin structure infections consistently demonstrated non-inferiority [73,74,75]. Only a single study reported superiority of tedizolid over linezolid in this indication, conducted in Japan [76].

Thirty-day mortality in infections caused by carbapenem-resistant *Acinetobacter baumannii* (CRAB) ranges from 23.5% to 66.4%, depending on patient- and disease-related factors. Mortality is significantly influenced by the presence of septic shock, moderate to severe thrombocytopenia, acute kidney injury requiring hemodialysis, high severity scores (APACHE II or SOFA), and admission to the intensive care unit. These findings are consistently supported across multiple studies [77,78,79].

Mortality in CRAB infections is strongly associated not only with the presence of bacteremia but also with the need for hemodialysis, a factor that may compromise the correct administration of antibiotic doses [80,81].

A promising therapeutic advance was the FDA approval in 2023 of sulbactam–durlobactam (XacDURO) for the treatment of hospital-acquired and ventilator-associated pneumonia caused by the *Acinetobacter baumannii–calcoaceticus* complex. This agent is considered an important step forward in reducing mortality rates from these healthcare-associated infections.

Similar carbapenem-resistance challenges are also associated with *Enterobacterales*. The use of polymyxins in combination with tigecycline or ceftazidime/avibactam with aztreonam has been linked to high mortality rates, which are further influenced by prior length of hospitalization and the presence of coinfections [82].

Importantly, antibiotic combinations have not consistently reduced mortality compared with monotherapy, as demonstrated in critically ill patients with carbapenem-resistant *Acinetobacter baumannii* bacteremia [83].

In Europe, an alarming increase has been observed in carbapenem-resistant *Escherichia coli* sequence type (ST) 13 isolates, as well as in New Delhi metallo-β-lactamase-1 (NDM-1)-producing *Providencia stuartii*, highlighting the urgent need for genomic surveillance and coordinated containment strategies [84].

The increasing resistance among *Enterobacterales* is estimated at an annual rate of approximately 5% [85]. Prevalence is consistently higher in Southern Europe, Southeast Asia, Africa, and the Western Pacific, compared with Northern Europe or North America [86].

A concerning observation is the increasing rate of gut colonization in otherwise healthy individuals, with 14% carrying ESBL- or carbapenemase-resistant strains, regardless of previous healthcare contact [87,88,89].

Furthermore, international travel plays a major role in dissemination, with individuals moving from low-resistance settings to high-prevalence regions at increased risk of colonization with ESBL or carbapenem-resistant *Enterobacterales* (CRE). This is well documented in systematic reviews and meta-analyses, showing that travel-acquired colonization significantly contributes to the global spread of resistance [90,91,92].

### 3.2. Future Possible Therapeutic Options

Antimicrobial resistance requires coordinated and clear decision strategies that include the rapid identification of new therapeutic options, the development of novel vaccines, fast and accurate etiological diagnostics, implementation of preventive measures and infection control strategies for healthcare-associated infections, as well as regional and national policies for monitoring antibiotic consumption in both the human and veterinary sectors.

Future therapeutic strategies focus on molecules with mechanisms of action distinct from currently used classes, designed to overpass existing bacterial resistance pathways. Among these, lasso peptides represent a promising option. Lariocidin, for example, targets the small ribosomal subunit by interacting with 16S rRNA and aminoacyl-tRNA, thereby altering ribosomally synthesized and post-translationally modified peptides through intramolecular cyclization, dehydration, and heterocycle formation. Preclinical data, including in vitro and in vivo models, suggest potent activity against Gram-positive bacteria (*Bacillus subtilis*), as well as Gram-negative bacilli such as *Escherichia coli*, *Acinetobacter baumannii*, and *Mycobacterium smegmatis* [93].

In addition, several naturally derived antibiotics are currently under investigation. Corallopyronin (CorA) has shown promise for the treatment of infections caused by resistant *Neisseria gonorrhoeae* and MRSA, while Clovibactin has demonstrated potent activity against MRSA and *Mycobacterium tuberculosis*. Chlorotonil A, a soil-derived metabolite, has exhibited efficacy against *Clostridioides difficile* and MRSA. Furthermore, bacteriophage therapy remains a potential option, particularly for the decolonization of vancomycin-resistant *Enterococcus faecium* (VRE) and MRSA [94].

Zosurabalpin is a tethered macrocyclic peptide (MCP) representing a novel antibiotic class that has shown promise against carbapenem-resistant *Acinetobacter baumannii*. Its mechanism of action involves blocking the lipopolysaccharide (LPS) transporter at the outer membrane, leading to intracellular accumulation of LPS and subsequent bacterial lysis [70,71]. However, the emergence of selective mutations in LPS or genetic alterations in genes encoding LPS synthesis (*lpxM*) or efflux regulators (*adeS* and *adeR*) highlights the potential risk of resistance, suggesting that zosurabalpin may need to be used in combination with other agents to preserve its efficacy [95,96].

Other innovative approaches are targeting specific multidrug-resistant pathogens. For *Neisseria gonorrhoeae*, Austrian researchers have identified a novel alkyl quinolone compound that selectively acts against gonococci without exerting adverse effects on human cells or other bacterial species [95]. Similarly, Swedish investigators are developing Debio 1453, a first-in-class inhibitor of the FabI enzyme, representing a mechanism of action distinct from currently available antibiotics and specifically designed for drug-resistant gonococcal infections [96].

Table 1 provides an overview of in-use antibiotics and selected novel antibiotics currently under investigation.

### 3.3. Strengths and Methodological Rigor

This review has several important strengths. First, it is based on a large number of studies, allowing for a more comprehensive synthesis of the available evidence than has previously been reported. The search strategy covered multiple databases and was conducted using predefined eligibility criteria, thereby minimizing the risk of missed studies and ensuring that only methodologically sound research was included.

### 3.4. Limitations

At the review level, despite our comprehensive search strategy, we cannot exclude the possibility of publication bias. The inclusion of only studies published in English may also have introduced language bias, potentially excluding relevant evidence published in other languages.

## 4. Conclusions

The remarkable adaptability of microorganisms to currently available antibiotics has drastically reduced effective therapeutic options worldwide. A major concern is the inability to adequately treat infections caused by XDR pathogens, with global deaths alarmingly projected to exceed 10 million annually by 2050. The urgent discovery of novel molecules with mechanisms of action distinct from those of existing antibiotics, alongside the development of alternative non-antibiotic therapies, may provide critical solutions to this escalating global health crisis.

## Figures and Tables

**Table 1:** Highlights of antibiotics for multidrug-resistant bacteria, actual agents, and novel therapeutic agents adapted from [97,98,99].

Antibiotic/Antibiotic Class	Mechanism of Action	Spectrum of Activity	Observation/Limitations
Tigecycline	Inhibits protein synthesis by binding to the bacterial 30S ribosomal subunit	ESBL, CRE (all classes including MBL), DTR *A. baumannii*	No activity against *P. aeruginosa*
Colistin	Disrupts bacterial cell membrane integrity by binding to LPS and phospholipids in the outer membrane of GNB bacteria	ESBL, CRE (all classes including MBL), DTR *P. aeruginosa*, DTR *A. baumannii*	Should be used in combination with one or more additional agents that highlights a susceptible MIC
Fosfomycin	Inhibits bacterial cell wall synthesis by targeting MurA enzyme	ESBL, CRE (all classes including MBL), DTR *P. aeruginosa*	Its use as monotherapy is not recommended
Cefiderocol	Siderophore cephalosporin: actively transported into the bacteria via iron transport systems	ESBL, KPC, MBL, AmpC β-lactamases, OXA-48 carbapenemase, DTR *P. aeruginosa*, DTR *A. baumannii*	
Ceftazidime– avibactam	Inhibits bacterial cell wall synthesis; avibactam inhibits β-lactamases, including KPC and OXA-48 carbapenemase	ESBL, KPC, AmpC β-lactamases, OXA-48 carbapenemase, DTR *P. aeruginosa*	No activity against MBL Important resistance rates in *A. baumannii* isolates
Ceftolozane– tazobactam	Inhibits bacterial cell wall synthesis; tazobactam inhibits β-lactamases	ESBL, DTR *P. aeruginosa*	No activity against carbapenemases-producing strains, DTR *A. baumannii*, AmpC β-lactamases
Imipenem– cilastatin– relebactam	Inhibits bacterial cell wall synthesis; relebactam inhibits KPC	ESBL, KPC, Relebactam may slighty enhance the activity of imipenem against OXA-carbapenemases, DTR *P. aeruginosa*	No activity against MBL producing strains
Carbapenems (e.g., meropenem, imipenem–cilastatin, ertapenem)	Inhibit bacterial cell wall synthesis by binding to PBPs	ESBL	No activity against carbapenemases, DTR *A. baumannii* or *P. aeruginosa* Ertapenem is inactive against *P. aeruginosa*
Meropenem– vaborbactam	Inhibits bacterial cell wall synthesis; vaborbactam inhibits KPC-producing β-lactamases	KPC, and ESBL	No activity against MBL- or OXA-type carbapenemases, DTR *P. aeruginosa* or *A. baumannii*
Ceftaroline	Binds with high affinity to penicillin-binding proteins (especially PBP2a in MRSA and PBPs 1–3), inhibiting peptidoglycan cross-linking and leading to cell wall weakening and lysis	Beta-hemolytic streptococci, Methicillin-resistant *Staphylococcus aureus*, Methicillin-resistant *Staphylococcus epidermidis*, *Streptococcus pneumoniae*, Viridans group streptococci	Partial antimicrobial activity against Vancomycin-resistant *Enterococcus*
Ceftobiprole	Forms stable complexes with PBPs, including PBP2a (MRSA) and PBP2x (penicillin-resistant *S. pneumoniae*), blocking peptidoglycan cross-linking and causing bacterial cell apoptosis	Beta-hemolytic streptococci, Methicillin-resistant *Staphylococcus aureus*, Methicillin-resistant *Staphylococcus epidermidis*, *Streptococcus pneumoniae*, Viridans group streptococci	No antimicrobial activity against Vancomycin-resistant Enterococcus
Oritavancin	Binds to D-Ala-D-Ala termini of peptidoglycan precursors, inhibiting transglycosylation and transpeptidation. It also disrupts membrane integrity and inhibits RNA synthesis	Beta-hemolytic streptococci, Methicillin-resistant *Staphylococcus aureus*, Methicillin-resistant *Staphylococcus epidermidis*, *Streptococcus pneumoniae*, Vancomycin-resistant *Enterococcus*, Viridans group streptococci	
Dalbavancin	Binds tightly to the D-Ala-D-Ala residues of peptidoglycan chains, preventing cell wall elongation and cross-linking	Beta-hemolytic streptococci, Methicillin-resistant *Staphylococcus aureus*, Methicillin-resistant *Staphylococcus epidermidis*, *Streptococcus pneumoniae*, Viridans group streptococci	No antimicrobial activity against Vancomycin-resistant *Enterococcus*
Omadacycline	Inhibits bacterial protein synthesis by binding to the 30S ribosomal subunit, blocking tRNA binding and peptide elongation	Beta-hemolytic streptococci, Methicillin-resistant *Staphylococcus aureus*, Methicillin-resistant *Staphylococcus epidermidis*, *Streptococcus pneumoniae*, Vancomycin-resistant *Enterococcus*, Viridans group streptococci	
Tedizolid	Binds to the 23S rRNA of the 50S ribosomal subunit, preventing the formation of the 70S initiation complex and thereby inhibiting protein synthesis	Beta-hemolytic streptococci, Methicillin-resistant *Staphylococcus aureus*, Methicillin-resistant *Staphylococcus epidermidis*, *Streptococcus pneumoniae*, Vancomycin-resistant *Enterococcus*, Viridans group streptococci	
Delafloxacin	Inhibits bacterial DNA gyrase and topoisomerase IV, enzymes essential for DNA replication, transcription, and repair	Beta-hemolytic streptococci, Methicillin-resistant *Staphylococcus aureus*, Methicillin-resistant *Staphylococcus epidermidis*, *Streptococcus pneumoniae*, Viridans group streptococci	No antimicrobial activity against Vancomycin-resistant *Enterococcus*
** Novel therapeutic agents **			
Darobactin	Binds to BamA	Gram-negative pathogens	Still in preclinical and early clinical development
Metazzobactam	Inhibits PBPs involved in bacterial cell wall synthesis	Gram-negative pathogens	Limited or no activity against carbapenemase-producing strains. Clinical development
Zosurabalpin	Blocks the lipopolysaccharide (LPS) transporter (LptB2FGC complex)	Carbapenem-resistant *Acinetobacter baumannii*	Active in preclinical and early clinical studies
Cefepime/Enmetazobactam	Cefepime: inhibits PBPs, disrupting peptidoglycan synthesis. Enmetazobactam: inhibits class A β-lactamases (including ESBLs)	Active against Enterobacterales (including ESBL-producers), some activity against Pseudomonas aeruginosa	not effective against carbapenemase producers. Approved in Europe (2024) for complicated urinary tract infections (cUTIs); phase III showed non-inferiority/superiority vs. piperacillin-tazobactam. Limited coverage against carbapenem-resistant strains
Cresomycin	Binds to the 50S ribosomal subunit, inhibiting protein elongation	Against macrolide-resistant Gram-positive bacteria	Still under investigation; clinical development status is early, with no large-scale trials completed yet
Lariocidin	Binds to the 16S rRNA of the small ribosomal subunit, interfering with aminoacyl-tRNA accommodation and protein synthesis	Gram-positive bacteria (e.g., *Bacillus subtilis*) and selected Gram-negative species (e.g., *Escherichia coli*, *Acinetobacter baumannii*, *Mycobacterium smegmatis*)	Preclinical stage

CRE: carbapenem-resistant *Enterobacteriaceae*; DTR: difficult to treat; ESBLs: extended-spectrum beta-lactamases; KPCs: *Klebsiella pneumoniae* carbapenemases; LPSs: lipopolysaccharides; MBLs: metallo-β-lactamases; MIC: minimal inhibitory concentration; PBPs: penicillin-binding proteins.

## Data Availability

No new data were created or analyzed in this study. Data sharing is not applicable to this article.
